# Androgen Receptor Could Be a Potential Therapeutic Target in Patients with Advanced Hepatocellular Carcinoma

**DOI:** 10.3390/cancers9050043

**Published:** 2017-05-05

**Authors:** Tatsuo Kanda, Koji Takahashi, Masato Nakamura, Shingo Nakamoto, Shuang Wu, Yuki Haga, Reina Sasaki, Xia Jiang, Osamu Yokosuka

**Affiliations:** 1 Department of Gastroenterology and Nephrology, Chiba University, Graduate School of Medicine, 1-8-1 Inohana, Chiba 260-8670, Japan; koji517@gmail.com (K.T.); nkmr.chiba@gmail.com (M.N.); nakamotoer@yahoo.co.jp (S.N.); gosyou100@yahoo.co.jp (S.W.); hagayuki@gmail.com (Y.H.); reina_sasaki_0925@yahoo.co.jp (R.S.); jxia925@yahoo.co.jp (X.J.); yokosukao@faculty.chiba-u.jp (O.Y.); 2 Department of General Surgery, The First Hospital of Hebei Medical University, Donggang Road No. 89, Shijiazhuang 050031, China

**Keywords:** androgen receptor, hepatocellular carcinoma, sorafenib

## Abstract

Hepatocellular carcinoma (HCC) is a male-dominant disease with poor prognosis. Sorafenib is the only approved systemic chemotherapeutic drug for patients with advanced HCC. Previous studies have shown that androgen and androgen receptor (AR) are involved in human hepatocarcinogenesis and the development of HCC. Here, we discuss the recent data on AR and HCC, and the combination of sorafenib and inhibitors of AR for advanced-HCC patients. Androgen-dependent and androgen-independent AR activation exist in human hepatocarcinogenesis. AR could directly control hepatocarcinogenesis and regulate the innate immune system to influence HCC progression. Combination of sorafenib with AR inhibitors might represent a potential treatment for patients with advanced HCC.

## 1. Introduction

Hepatocellular carcinoma (HCC) is one of the poor-prognosis cancers [1,2]. In Japan, HCC is the major cancer among primary liver cancers, which have 5- and 10-year survival rates of 34% and 16%, respectively [3]. HCC mostly occurs in patients with cirrhosis. It is not easy to cure HCC by surgical resection other than liver transplantation [4]. In patients with advanced HCC, sorafenib is the only approved systemic chemotherapeutic drug, and new treatment options are eagerly awaited [1].

To surpass the treatment with sorafenib alone for advanced HCC, new treatments have been developed in recent years [2,5,6]. Histone deacetylase inhibitor resminostat plus sorafenib was safe and showed early signs of efficacy for advanced HCC patients progressing on sorafenib-only treatment [5]. Sorafenib plus hepatic arterial infusion chemotherapy with cisplatin achieved favorable overall survival when compared with sorafenib alone for advanced HCC patients [6]. Regorafenib was also shown to provide survival benefit in advanced HCC patients progressing on sorafenib treatment [2].

HCC is one of the male-dominant cancers [7]. We and others have reported that male sex hormone androgen and androgen receptor (AR) are involved in human hepatocarcinogenesis and the development of HCC [8,9,10,11,12]. AR antagonists such as flutamide and bicalutamide have been used for prostate cancer for many decades, and new AR antagonists are also under development [13]. Herein, AR and HCC will be discussed. We also describe the combination treatment of sorafenib and inhibitors of AR for patients with advanced HCC.

## 2. AR and AR Signaling

**Figure 1:**
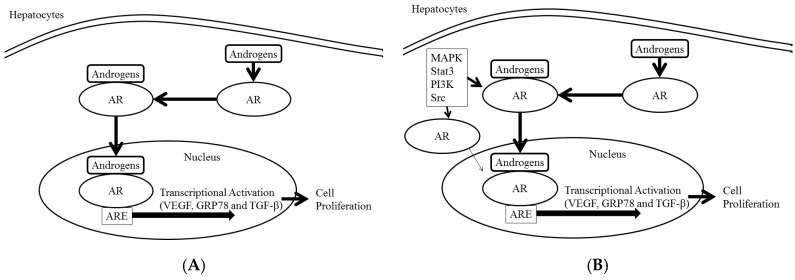
Androgen-dependent and androgen-independent androgen receptor (AR) activation in human hepatocarcinogenesis. (**A**) Androgen-dependent signaling. (**B**) Androgen-independent signaling. Phosphorylation of mitogen-activated protein kinase (MAPK), signal transducer and activator of transcription 3 (Stat3), AKT serine/threonine kinase 1 (Akt) and Proto-oncogene tyrosine-protein kinase (Src) activates AR. VEGF, vascular endothelial growth factor; GRP78, glucose-regulated protein 78 kDa; TGF-β, transforming growth factor, beta 1; PI3K, phosphatidylinositol-4,5-bisphosphate 3-kinase catalytic subunit alpha.

Androgens act through AR, a 110-kDa ligand-inducible nuclear receptor (Figure 1A) [14]. The classical steroid receptors such as AR, estrogen receptor, progesterone receptor, glucocorticoid receptor and mineral corticoid receptor are grouped as type 1 nuclear receptors. AR has four functional domains: NH_2_-terminal transactivation domain, DNA-binding domain (DBD), hinge region and ligand-binding domain (LBD).

AR regulates the expression of target genes that have androgen response elements (AREs) (Figure 1A) [14,15]. AREs exist in the promoter region of vascular endothelial growth factor (VEGF) [8] and glucose-regulated protein 78 kDa (GRP78) [9], and they play a role in the growth of human hepatocytes. Transforming growth factor, beta 1 (TGF-β1) transcription is also activated by androgen and AR complex in hepatocytes [16,17]. This transcriptional activation function of AR is important in the normal sexual development of the male gender as well as the progression of cancer [8,14,18].

AR co-regulators also influence a number of functional properties of AR, including ligand selectivity and DNA binding capacity [14]. Oncogenes such as erb-b2 receptor tyrosine kinase 2 (ERBB2) and HRas proto-oncogene, GTPase (HRAS) increase mitogen-activated protein kinase signaling, which can cause ligand-independent activation of AR (Figure 1B) [19,20]. There is a cross-talk mechanism between growth factor signaling and androgen in prostate development, physiology, and cancer [20]. Ligand-independent activation of AR pathways also plays a role in human HCC and pancreatic cancer progression [8,21].

The activation of Src kinase is involved in the ligand-independent activation of AR [22]. Two UDP-glucuronosyltransferases (2B15 and 2B7) are also involved in inactivation of androgens, and may have a major role in persons that is null genotype of UGT2B17 [23]. Hepatitis B X (HBx) also augmented AR activity by enhancing the phosphorylation of AR through HBx-mediated activation of the c-Src kinase signaling pathway in human hepatocarcinogenesis [11,24].

## 3. AR and HCC

Human HCC and normal liver express AR [7,10,25]. Hepatitis B virus (HBV) and hepatitis C virus (HCV) are two major causes of HCC. AR signaling is involved in human HCC associated with HBV and HCV [26]. AR signaling should be involved in hepatocarcinogenesis to some extent, irrespective of the cause of human and mouse HCC [27]. As androgen and AR-signaling are associated with the development of steatosis [28], AR may be associated with HCC that is related to non-alcoholic steatohepatitis.

Increased expression of variant transcripts from the AR gene (ARVs) has been shown to be involved in the development of castration-resistant prostate cancer [29]. The expression of ARVs was observed in the liver and may be involved in hepatocarcinogenesis [30]. AR variants may also lead to resistance to HCC antiandrogen therapy in the liver.

## 4. AR and Sorafenib in the Treatment of HCC (Table 1)

At present, sorafenib is the only approved drug for systemic chemotherapy of HCC. We observed that sorafenib-induced apoptosis was enhanced by the inhibition of AR and GRP78 in human hepatoma cell lines [9]. Sorafenib also inhibits AR activation induced by HBx in vitro and in vivo [31]. Of interest, this AR-targeting ability of sorafenib was not mediated by its well-known kinase inhibitory activity; however, this ability of sorafenib was achieved by enhancing the activity of K-box region and MADS-box transcription factor family protein (SHP-1) tyrosine phosphatase [31]. There are contrary opinions concerning hepatic AR and the effect of sorafenib, namely that hepatic AR suppresses HCC metastasis through modulation of cell migration and anoikis [30,32,33]. Natural killer (NK) cells suppress HCC; and interleukin 12 (IL12A), one of the NK cell stimulatory factors, plays a role in the activation of NK cell function [34,35]. In NK cells, AR could suppress IL12A expression at the transcriptional level, resulting in repressing the efficacy of NK cell cytotoxicity against HCC [34]. Sorafenib treatment interacts with AR and enhances IL12A signals [34]. AR could regulate the innate immune system to influence HCC progression [34,36,37]. Although AR suppresses HCC metastasis at late stage [28,32,33,37], androgen and the AR axis maintain and promote cancer cell stemness through activation of Nanog in HCC [38]. 

## 5. Conclusions

We have already reviewed clinical trials targeting androgen in HCC [25]. However, the previous reports demonstrated that anti-androgen therapies did not show any survival benefits in advanced HCC patients [39,40]. That might be considered to be attributed by the lower expression of AR and androgen-independent AR activation mechanism in the advanced HCC. A recent review [13] described phase I/II clinical trials of the androgen antagonist enzalutamide with or without sorafenib for advanced HCC that are currently underway. Enzalutamide binds to the AR with greater relative affinity than the clinically used antiandrogen bicalutamide, reduces the efficiency of its nuclear translocation, and impairs both DNA binding to androgen response elements and recruitment of coactivators [41]. The combination of sorafenib and enzalutamide is a potentially new approach for the treatment of castration-resistant prostate cancer [42]. This combination may present a potential treatment for patients with advanced HCC. In prostatic cancer cells with downregulated AR expression by short interfering RNA, treatment with sorafenib increased apoptosis in an additive manner [43], suggesting that there might be a potential to use inhibitors of AR in HCC as an adjuvant therapy option for sorafenib-resistant HCC patients. Moreover, immune checkpoint inhibitors such as programmed cell death 1 (PD-1), programmed cell death ligand 1 (PD-L1), or cytotoxic T-lymphocyte-associated protein 4 (CTLA-4) are now undergoing clinical trials, and they may open new doors for the treatment of HCC [44]. In this new era, AR could control NK cell function and may be a more attractive target. In conclusion, recent advances regarding AR in HCC have been described. AR is an attractive target with or without anti-cancer drugs in HCC, one of the male dominant diseases.

## Figures and Tables

**Table 1:** Molecular targets during anti-cancer drug treatment for hepatocellular carcinoma (HCC) through androgen receptor (AR).

References	Targets	Effects of Anti-Cancer Drugs
Jiang et al. [9]	GRP78	Knockdown of GRP78 and AR enhances apoptosis induced by sorafenib in human hepatoma cells.
Wang et al. [31]	SHP-1	Sorafenib inhibited HBx-enhanced AR activity by activating SHP-1 phosphatase in HBx-transgenic mice.
Shi et al. [34]	IL12A	Sorafenib interacts with AR and enhances IL12A signals.
Shi et al. [36]	ULBP2	By suppressing AR, cisplatin could up-regulate cytotoxicity of NK cells to target HCC.
Ma et al. [28]	p-p38, NFκB, MMP9	Addition of sorafenib improved HCC survival of L-AR^−^^/y^ mice.
Xu et al. [33]	miR-367	Combining miR-367-3p with Sorafenib showed better efficacy of suppressing HCC cell invasion by altering AR signals in vitro and in vivo.

GRP78, glucose-regulated protein 78 kDa; SHP-1, K-box region and MADS-box transcription factor family protein; HBx, hepatitis B x; IL12A, interleukin 12A; ULBP2, UL16-binding protein 2; p-p38, phosphorylation of p38 kinase; NF-κB, nuclear factor kappa B; MMP9, matrix metalloproteinase 9.
